# General principles and escalation options of immunotherapy in autoantibody-associated disorders of the CNS

**DOI:** 10.1186/s42466-019-0037-x

**Published:** 2019-10-01

**Authors:** Ilya Ayzenberg, Simon Faissner, Laura Tomaske, Daniel Richter, Volker Behrendt, Ralf Gold

**Affiliations:** 1 Department of Neurology, St. Josef-Hospital, Ruhr-University Bochum, Bochum, Germany; 2 Department of Neurology, Sechenov First Moscow State Medical University, Moscow, Russia

## Abstract

Autoimmune diseases associated with antineuronal and antiglial autoantibodies (Abs) is one of the most rapidly expanding research fields in clinical neuroimmunology, with more than 30 autoantibodies described so far. Being associated with a wide range of clinical presentations these syndromes can be diagnostically challenging. Surface or intracellular antigen localizations are crucial for the treatment response and outcome. In the latter Abs are mostly of paraneoplastic cause and tumor management should be performed as soon as possible in order to stop peripheral antigen stimulation. Immunotherapy should be started early in both groups, before irreversible neuronal loss occurs. Despite serious prognosis, aggressive therapeutic approaches can be effective in many cases. In this article we review main pathogenic mechanisms leading to Abs-related syndromes and describe standard as well as emerging strategies of immunotherapy, including tocilizumab and bortezomib. Several special therapeutic approaches will be illustrated by clinical cases recently treated in our department.

## Introduction

Autoimmune diseases associated with antineuronal and antiglial autoantibodies (Abs) is one of the most rapidly expanding research fields in clinical neuroimmunology. Autoantibodies targeting more than 30 specific antigens in the central nervous systems (CNS) have been identified so far and several new candidate antigens are reported every year [1]. Intracellular or surface target protein localisation and in the last case its function often determine clinical presentation as well as key immunological mechanisms and accordingly preferable therapeutic approaches. Rarity and clinical diversity of the Abs-associated syndromes, lack of specific clinical features and partly overlapping symptoms are challenging for diagnostics. Studies on management are generally limited and large double-blind clinical trials have been conducted in neuromyelitis optica spectrum disorder (NMOSD) only [2]. Experience gathered from NMOSD management is useful, however, cannot be automatically transferred to other diseases associated with antineuronal Abs due to pathogenic differences. Treatment recommendations are mostly based on retrospective case series and expert opinions. Although several generally accepted therapy principles have been elaborated in the last decades, an individual strategy is often required, especially in rare syndromes and/or therapy refractory cases. Here, we discuss principles of management of Abs-associated CNS-diseases and describe several challenging cases, recently treated in our department.

### Abs directed towards intracellular antigens

The Abs targeting intracellular proteins are mostly (with an exception of Abs against GAD-65 (glutamate decarboxylase)) of paraneoplastic origin and associated with tumors, expressing neuronal antigens or directed against ubiquitous nuclear antigens released due to continuous cell death in progressive tumors. It is believed, that the anti-tumor immune response partly supresses tumor growth, thereby resulting in a breakthrough of the immunological tolerance with development of an autoimmune disease. This hypothesis explains well an early manifestation of neurological syndromes, in 2/3 of cases prior to cancer diagnosis [3]. The increasing number of reports on paraneoplastic syndromes (PNS) under tumor therapy with checkpoint inhibitors also supports this theory [4]. Due to an intracellular Ag-localization onconeuronal Abs are probably not involved into pathogenesis, however serve as an important diagnostic epiphenomenon. In contrast, a T-lymphocyte mediated immune response results in a rapid irreversible neuronal loss within several weeks or months leading to permanent deficits [5]. Accordingly, the stroke principle “time is brain” is also relevant in PNS. An early tumor treatment is the most important management step. Prompt removal or depletion of tumor cells, as a source of peripheral antigen stimulation, decreases activity of the autoimmune response and can alone be sufficient for stabilization or even improvement of the PNS [6–8].

Despite an overall clinical heterogeneity, several red flags can be helpful for the early diagnostics of PNS:

- mostly subacute manifestation with continuous progression over weeks or months;
- disease manifestation at age > 45 years (with a few exceptions, e.g. opsoclonus-myoclonus syndrome in children, teratoma-associated anti-NMDAR (N-methyl-D-aspartate receptor) encephalitis in young females or Ma2-Abs in men < 50 years);
- typical clinical manifestations (without a plausible alternative explanation): subacute cerebellar degeneration, brainstem or limbic encephalitis, opsoclonus-myoclonus syndrome, stiff-person syndrome, sensory neuronopathy, neuromyotonia or Lambert-Eaton syndrome;
- sometimes multilocular manifestation (e.g. in both central and peripheral nervous system).

Cerebrospinal fluid analysis may reveal non-specific inflammatory changes and MRI is mostly normal or may rarely demonstrate characteristic symmetric inflammatory lesions (e.g. in the cerebellum in subacute cerebellar atrophy) in early stages and atrophy in late stages.

It is generally accepted that the first line immunotherapy should be initiated early, preferably within a few weeks after disease manifestation, even before definite identification of the underlying tumor [9–11]. In most cases adequate immunosuppressive therapy does not affect tumor diagnostics (exception: steroid therapy in suspected lymphoma) as well as tumor outcome [12].

### Tumor detection

Since the autoimmune response suppresses tumor growth, the tumors are often initially small and asymptomatic [3, 13]. PNS are mostly associated with lung cancer (small cell > > non-small cell lung cancer), ovarian and breast cancer, Hodgkin and non-Hodgkin lymphoma or thymoma [3, 14]. Presence of several antineuronal Abs in one patient is not rare and helps to narrow further diagnostic workup [15, 16]. In special situations a testicular cancer (in men younger than 50 years with Ma2-Abs) and neuroblastoma (in children with opsoclonus-myoclonus syndrome or rarely Hu-Abs) should be considered [17, 18]. Depending on suspected malignancy, a targeted diagnostic approach should be performed [13]. If standard diagnostic work-up remains negative, a whole-body FDG-PET/CT can be helpful [19]. If FDG-PET/CT is negative, tumors with low proliferation rates (e.g. differentiated teratomas, neuroendocrine tumors) or non–metastatic skin cancers should be considered. Rarely even thorough first tumor screening may remain negative. Further diagnostic assessment is recommended within 3 months and then every 6 months for a period of 4 years [13].

### Abs directed toward surface antigens

In contrast, **Abs targeting surface antigens** are only facultative of paraneoplastic origin and often occur as a primary autoimmune disorder, especially in younger patients. Loss of function of surface target proteins, including synaptic receptors, ion channels or associated membrane proteins, explain direct pathogenic significance of autoantibodies. Interestingly, the clinical presentation is often similar to genetic disorders with mutations in the same target protein (e.g. focal seizures in LGI1-Abs (leucine rich glioma inactivated 1) and familial temporal lobe epilepsy with mutation in the LGI1 gene [20, 21]. Several immunologic mechanisms have been described in this subgroup of Abs-associated diseases so far [22]:

- receptor cross-linking and internalization, resulting in a decreased receptor density in the synapse;
- direct agonistic or antagonistic action on the receptor itself;
- activation of the complement cascade or antibody-dependent cell-mediated cytotoxicity (with irreversible deficits).

The respective IgG-subclass may contribute to the pathogenesis and should be considered by choosing the best suited therapy. Although being usually of IgG1 subclass, several Abs (e.g. LGI1-, CASPR2- (contactin associated protein 2), IgLON5- etc.) predominantly belong to the **IgG4 subclass**. Being able to exchange half-molecule (so called “Fab-arm exchange”) IgG4 are bispecific and functionally monovalent [23]. Moreover, they have low affinity for the Fcγ receptor. Accordingly, autoantibodies of IgG4 subclass cannot induce cross-linking, complement activation or cell-mediated cytotoxicity. Abs-related interference of the ligand-receptor interaction has been supposed as one of possible pathogenic mechanisms in IgG4-related neurologic disorders [24]. Yet a combination of autoantibodies from several subclasses can be found in most cases, making interpretation of the precise pathogenic mechanisms difficult.

Due to a direct pathogenic role of autoantibodies, Abs-depleting immunotherapies, including apheresis in the acute stage and B-cell targeting long-term therapies are effective in most cases. If treated appropriately, an outcome in case of Abs targeting surface proteins is considerably better, comparing to classical PNS, associated with immune responses against intracellular antigens. An early start of the first line therapy is associated with a better long-term outcome in those syndromes [25, 26].

### General principles of immunotherapy

Main therapy regimens are summarized in the Table 1. In both paraneoplastic and primary autoimmune forms **first line treatment** at the acute stage usually includes either corticosteroid pulse therapy (e.g. 5 × 1000 mg methylprednisolone IV, in some cases followed by oral tapering) or intravenous immunoglobulin G (IVIG, e.g. 0.4 g/kg bodyweight for 5 days). If no sufficient improvement can be achieved, an early escalation to immunoadsorption or plasma exchange should be undertaken. IVIG treatment and apheresis therapies are especially effective in case of Abs directed against surface antigens compared to intracellular antigens [9, 27–29]. If no improvement occurs, an early (up to 2 weeks after the primary treatment) escalation to cyclophosphamide (as a short-term high-dose treatment with 750–1000 mg/m^2^ IV) or rituximab (e.g. 500 mg – 1000 mg IV) can be performed [9, 30, 31]. Treatment of paraneoplastic cases is often challenging and tumor therapy is most important for stabilization of the neurological deficits [6–8].

**Table 1:** Standard therapeutic approach and escalation therapies

First-line therapies	
Methylprednisolone	1000 mg/day for 5 days, if needed with oral tapering
Intravenous immunoglobulin	0.4 g/kg/day over 5 days
Plasma exchange or immunoadsorbtion	5–7 cycles
* +Tumor therapy in paraneoplastic cases as soon as possible!*	
Escalation immunotherapies^§^	
Rituximab	Initially 500–2000 mg IV, followed by 250–1000 mg every 6 months or depending on B-cell repopulation*
Cyclophosphophamide	Induction with 750–1000 mg/m^2^ of BSA (e.g. 300–350 mg/m^2^/d over 3 days), followed by 500–750 mg/m^2^ of BSA every 4 weeks^#^
Further long-term immunotherapies^§^	
Intravenous immunoglobulin	1 g/kg body weight every 4–6 weeks IV, alternatively subcutaneously in equivalent dose (home setting)
Oral immunosuppressive drugs alone or in combination with prednisolone	
Azathioprine	2–3 mg/kg/d
Methotrexate	7.5–20 mg/week
Mycophenolate mofetil	1000–2000 mg/kg/d
Reserve therapies in refractory disease course	
Tocilizumab	8 mg/kg every 4 weeks
Bortezomib	1–2 cycles with 1.3 mg/m^2^/cycle s.c., administered on days 1, 4, 8, 11, followed by other long-term therapy.

*§Treatment duration depending on the individual relapse risk in different diseases.*

*Consider re-infusion already by beginning repopulation. Intervals can be usually prolonged in case of sustained depletion and clinical stabilization in patients > 50 years old and/or after several years of rituximab therapy

*#Absolute dose depends on leucocyte nadir. Due to toxicity a lifetime cumulative dosage is limited. Accordingly intervals can be prolonged or therapy can be switched in case of clinical stabilization.*

Depending on the risk of further relapses or progression a limited (e.g. for 1–2 years by teratoma-associated NMDAR-Abs encephalitis) or a life-long immunotherapy can be required (Table 1). **Long-term treatment** usually includes oral immunosuppressants (e.g. azathioprine 2–3 mg/kg/d, mycophenolate mofetil 250–1000 mg b.i.d. as a monotherapy or combined with oral steroids) or rituximab (e.g. 500 mg every 6 months). In some syndromes regular IVIG courses (usually 1 g/kg bodyweight every 4 to 8 weeks) can be effective (e.g. stiff-person syndrome, myelin oligodendrocyte glycoprotein (MOG) - Abs associated disease, IgLON5-syndrome).

Regular monitoring of both clinical (e.g. cognitive deficits, frequency and severity of seizures, spasticity, degree of ataxia etc.) and paraclinical (e.g. MRI-changes, intraindividual changes of Abs-titers, especially in CSF, or epileptiform activity in Video-EEG monitoring) disease activity is critically important in order to evaluate therapy responsiveness. We recommend an early first follow-up examination within 1–3 months after initiation of the immunotherapy. Further follow-up intervals can be extended and should be performed every 6–12 months, depending on the clinical entity and individual course of the disease.

### Outcome

Outcome in classical paraneoplastic syndromes is usually poor with a relative exception of Ma2-Ab-associated encephalomyelitis, in which approx. 30% of patients experience improvement after adequate tumor treatment and immunotherapy [32]. In contrast, patients with Abs targeting surface antigens have a much better prognosis, except for those with additional onconeural Abs [33, 34]. Especially in case of Abs-mediated cross-linking and internalization of the target–receptor-antibody complex together with Abs no or only minor neuronal loss occurs [35, 36]. This mechanism explains good or even complete recovery in about 75% of patients with NMDAR-encephalitis [37]. Still therapy refractory cases occur also in this group [38, 39]. It is supposed that main reasons might be compartmentalization of the immune response in the CNS (e.g. in NMDAR-encephalitis) and non-responsiveness of long-lived CD20-negative Abs-producing plasma cells to classical immunosuppressive agents and rituximab.

### Escalation therapies targeting plasma cells

In therapy refractory cases with a rapid ongoing clinical deterioration, prompt escalation to therapies directly targeting Abs-producing plasma cells can be critically important. Two of the most promising and already tested options include the anti-IL-6 receptor monoclonal antibody tocilizumab and the proteasome inhibitor bortezomib.

In NMOSD, three new substances are currently under investigation: the anti-IL-6 receptor antibody satralizumab (2nd generation substance following tocilizumab), the anti-CD19 monoclonal antibody inebilizumab and the anti-C5 complement factor directed antibody eculizumab. Belimumab, a monoclonal antibody targeting B-cell activating factor (BAFF), could also be potentially effective, however there is no real-life clinical experience with this substance in neurological diseases published so far.

### Tocilizumab

Tocilizumab is a humanized monoclonal antibody, targeting both soluble and membrane bound IL-6-receptor, approved in rheumatoid arthritis and juvenile idiopathic polyarthritis and giant cells arteritis. The IL-6 pathway plays an important role in various autoimmune diseases, being involved into differentiation of Abs-producing B-cells as well as IL-17 producing T-helper cells and IL-21-producing CD8+ T-cells [40, 41]. In NMOSD a specific CD19^int^CD27^high^CD38^high^CD180^−^ plasmablast subpopulation has been reported to produce AQP4 (aquaporin4)-Abs in an IL-6 dependent manner [42]. In 2013 we reported for the first time effective treatment of rituximab-refractory NMOSD patients with tocilizumab (8 mg/kg every 4 weeks) [43]. Both relapse activity and AQP4-Abs titer in serum decreased without serious side effects, with the longest therapy duration of 8 years in one patient so far. In some of these patients AQP4-Abs are not detectable any more (unpublished data).

In a recent phase 3 clinical trial satralizumab, a new long-circulating humanized monoclonal antibody targeting the IL-6 receptor, demonstrated impressive efficacy, especially in seropositive NMOSD [2]. Tocilizumab was also effective in refractory cases of contactin-associated protein-like 2 and NMDAR-encephalitis [39, 44]. Recently we started tocilizumab in two patients with an aggressive and therapy resistant MOG-Abs positive NMOSD. Previous therapies including azathioprine and rituximab in combination with oral prednisolone (20–30 mg/d) were not effective and patients developed multiple relapses approximately every 8 weeks. No further relapses occurred during the last months on tocilizumab therapy, however further and longer observations are needed (unpublished data).

Taken altogether, we suppose that tocilizumab is a promising second-line therapy in syndromes with Abs targeting surface proteins. Tocilizumab should be given in a dose of 8 mg/kg every 4 weeks. As previously demonstrated, prolonged intervals are associated with further relapse activity, at least by AQP4-Abs positive NMOSD [45]. Based on a large rheumatologic experience this therapy option is relatively safe in our region [46]. However, neutropenia, thrombocytopenia, elevated liver enzymes and hypercholesterinemia may rarely occur. Risk of infections is increased and should be thoroughly clinically monitored, as impaired IL-6 signaling results in usually normal C-reactive protein and normal body temperature even despite systemic infection.

### Bortezomib

Bortezomib is a selective reversible inhibitor of the enzymatic activity of the 20S proteasome subunit, approved for the treatment of multiple myeloma [47, 48]. Due to a high metabolic activity professional Abs-producing plasma cells are especially susceptible to proteasome blockade. Mechanistically, bortezomib causes an aberrant degradation and accumulation of defective ribosomal products in the endoplasmic reticulum. At the same time bortezomib decreases the degradation of the antiapoptotic nuclear factor kB (NF-kB) inhibitor, enhancing apoptosis in both myeloma and plasma cells. Furthermore, decreased activity of the NF-kB pathway results in a profound suppression of proinflammatory cytokines such as tumor necrosis factor alpha (TNF-α), IL-1β, IL-6, reduction of T-cell activation and induction of apoptosis in already activated and proliferating T-cells [49, 50]. Reduced protein degradation limits presentation of autoantigens to the immune system. Moreover, bortezomib impairs maturation of professional Ag-presenting dendritic cells (DC) and subsequently DC-mediated T-cell stimulation, especially demonstrated for alloreactive T-cells [51, 52].

Bortezomib has been successfully used in several rheumatologic diseases, including rheumatoid arthritis and refractory systemic lupus erythematosus [53, 54]. In the latter bortezomib induced a transient decrease of anti-ds-DNA-Abs. However, Abs recurred after bortezomib withdrawal, due to a rapid repopulation of plasma cells [55]. Accordingly, a combination of bortezomib with rituximab seems to be essential for a sustained response in chronic autoimmune diseases. Interestingly, in vitro data demonstrate that bortezomib-induced impaired intracellular degradation of CD20 results in its upregulation on the surface of lymphoblastoid B-cells [56]. Thus, it may increase the therapeutic effect of rituximab, supporting the rationale for a combination therapy with both medications. The small size and good permeability through the blood-brain barrier is an important advantage of bortezomib in neurological conditions, compared to large Abs-molecules, especially in case of intrathecal compartmentalization of the autoimmune response.

Experience in neurological diseases is limited. In 2016 we reported the first two patients with severe NMDAR-encephalitis responsive to bortezomib [57]. Our first patient, previously mechanically ventilated over 7 months and refractory to IVIG, corticosteroids, plasma exchange, rituximab and cyclophopsphamide, improved almost completely (except for mild cognitive deficits) under bortezomib. A second patient improved initially well under plasma exchange and rituximab, however relapsed 20 months later despite complete B-cell depletion. She was refractory to all first line therapies, including plasma exchange and achieved significant improvement after initiation of bortezomib mono-therapy only. Several further case reports and case series confirmed bortezomib as a promising escalation therapy in NMDAR-encephalitis recently [58–60].

Similar efficacy in other syndromes is likely associated with immune response against membrane antigens. Recently we reported the first positive experience in myasthenia gravis. A patient with anti-muscle-specific tyrosine kinase (MuSK)-Abs, previously non-responsive to high-dose IVIG, apheresis therapy, steroids and rituximab showed rapid and significant clinical improvement upon bortezomib therapy [61]. A phase IIa clinical trial investigating efficacy of bortezomib in myasthenia gravis, lupus erythematosus and rheumatoid arthritis is ongoing [62].

**Fig. 1:**
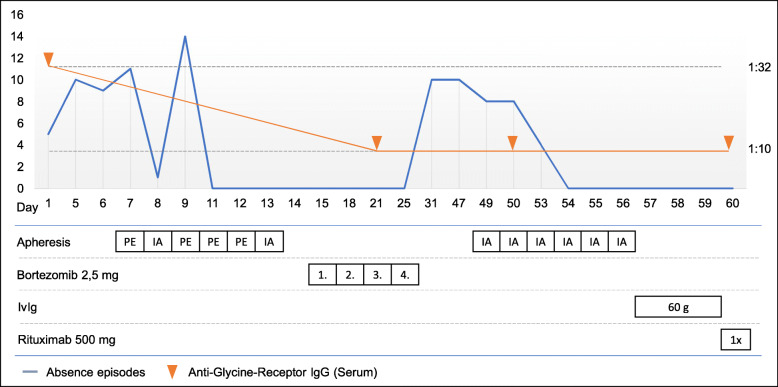
Frequency of absence episodes and titers of anti-glycine receptor-Abs in relation to the course of therapy. Please note that the timeline on the x-axis is nonlinear

Here, we describe a 20-year old man who developed anti-glycine receptor mediated epilepsy and was successfully treated with a combination therapy of rituximab and bortezomib (Table 2 , Fig. 1).

**Table 2:** Case Box 1

A 20-year-old man was admitted to our department due to generalized epileptic seizures since 13 months. In addition, he complained about absence episodes and myoclonic twitching up to 10-15 times a day. Previous diagnostic work-up revealed minimal swelling of the left amygdala without any further relevant abnormalities. Generalized seizures could be completely controlled with lamotrigine and brivaracetam, however absence episodes and myoclonic twitching persisted. Assuming possible autoimmune epilepsy cortisone therapy (a total of 5 cycles of 5 g methylprednisolone every 4 weeks) had been tried without any improvement and the patient was referred to our department for a second opinion. Diagnostic work-up revealed a granulocytic pleocytosis in the CSF as well as anti-glycine receptor antibodies in serum (1:32) while CSF anti-glycine receptor antibodies were negative. Herein we performed combined apheresis therapy with four cycles of plasma exchange (PE) and two cycles of immunoadsorption (IA), followed by a cycle of proteasome inhibitor bortezomib (4 x 2.5 mg). This led to dramatic improvement of seizure frequency and a drop of anti-glycine receptor antibody titers to 1:10. Myoclonic twitches were absent, however shortly after discharge the frequency of absence episodes rose again. Therefore, a second cycle of immunoadsorption followed by 60 g of intravenous immunoglobulins was performed. The absence episodes decreased again and did not occur afterwards. Due to the suggestion of a relapsing course of the disease rituximab was initiated as long-term therapy

**Fig. 2:**
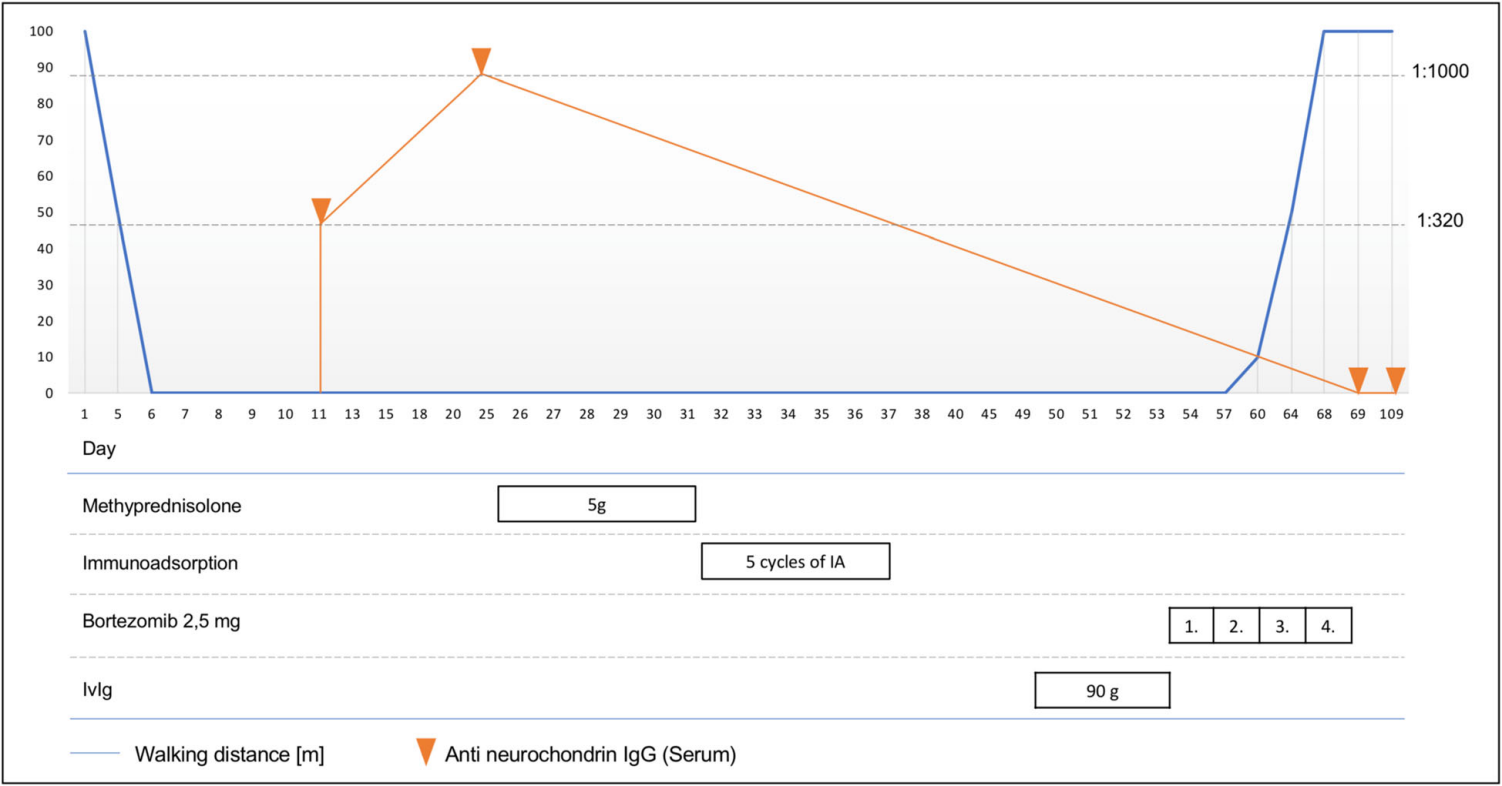
Walking distance and anti-neurochondrin-Abs titers in relation to the course of therapy. Please note that the timeline on the x-axis is nonlinear

Suppression of humoral autoimmunity explains many, but not all effects of proteasome inhibitors. Recently we observed significant clinical improvement under bortezomib in a patient with severe subacute cerebellar ataxia associated with Abs targeting neurochondrin and Delta notch-like epidermal growth factor-related receptor (DNER) (Table 3 , Fig. 2). DNER is a transmembrane protein, so that a direct pathogenic role of Abs cannot be excluded [63]. Still, previous studies could not confirm efficacy of the Abs-depleting therapy in this obligate paraneoplastic syndrome. Usually, it has a progressive course with irreversible deficits and poor functional outcome despite plasma exchange therapy [64]. Neurochondrin is located intracellularly and an antigen-specific T-cell response, but not autoantibodies, seems to be pathogenically relevant [65]. Interestingly, bortezomib administration also resulted in stabilization or even improvement in 10 patients with chronic inflammatory demyelinating polyneuropathy, classically histopathologically characterized by macrophage and T-lymphocyte infiltration [66]. None of these patients had specific nodal or paranodal autoantibodies. If bortezomib, due to its pleiotropic effects, could be effective in other diseases associated with a T-cell mediated immunity and intracellular antigen localization, remains highly speculative and should be investigated in the future. However, if effective, it would be an important treatment option in otherwise refractory classical paraneoplastic syndromes.

**Table 3:** Case box 2

A 54-year-old male was transferred to our department due to severe dysarthrophonia, double vision, nystagmus and ataxia in the last 7 weeks. The onset of symptoms was subacute over a few days. Brain MRI revealed an early cerebellar atrophy, consistent with aggressive course of subacute cerebellar degeneration. Routine CSF-analysis revealed 9 lymphocytic cells/μl, an elevation of total protein levels to 57.5 g/ml and CSF-specific oligoclonal immunoglobulin G (IgG) bands. Initial standard serum screening for antineuronal Abs was negative. Extensive analysis demonstrated autoantibodies against neurochondrin (titer of 1:1,000) in serum and autoantibodies against Delta/Notch-like Epidermal Growth Factor-Related Receptor isolated in CSF only (titer of 1:100). The following comprehensive diagnostic work-up including PET-CT detected a hypermetabolic area in the left parotid gland, diagnosed as Warthin´s tumor after biopsy. Other reasons for a paraneoplastic origin were not detected. First-line therapies, including steroid pulse (5 g of methylprednisolone), immunoadsorption (8 cycles) and IVIG (1 g/kg body weight) were non-effective and the patient deteriorated further. Due to a severe gait ataxia the patient could not walk without a both-sided assistance anymore. Severe nystagmus, double vision and dysarthrophonia made communication almost impossible. For symptomatic treatment, fampridine (20 mg per day) was started, resulting in a slight improvement of the dysarthrophonia only. Following, we performed a therapy cycle with bortezomib which surprisingly led to substantial improvement several days after therapy initiation. One week later the patient was able to walk without help and even climb stairs again. In a follow-up investigation two months later all symptoms had substantially improved. Neurochondrin- and DNER-Abs in serum were negative

Sensory axonal polyneuropathy is one of the most important limiting side effects of bortezomib, reaching a prevalence of 30–60% by high-dose treatment in multiple myeloma [67, 68]. In autoimmune conditions usually lower doses are sufficient. Further serious adverse events include pancytopenia, congestive heart failure, pulmonary edema, renal failure, gastrointestinal bleedings or infectious complications [69].

### Future promising options

Besides the above mentioned satralizumab, two further medications, targeting humoral autoimmunity and being currently investigated in NMOSD, could be promising in other Abs-mediated CNS diseases. Eculizumab is a humanized monoclonal IgG4 antibody, inhibiting cleavage of C5 complement factor into proinflammatory cell-activating C5a and lytic terminal complex component C5b. It is approved for the treatment of paroxysmal nocturnal hemoglobinuria, atypical hemolytic uremic syndrome and refractory myasthenia gravis [70, 71]. Recently, a successful phase 3 study of eculizumab in AQP4-Abs positive NMOSD has been reported [72]. Eculizumab could be an interesting escalation therapy in other diseases, associated with complement activating IgG1-Abs. Despite its poor blood-brain-barrier permeability (serum: CSF ratio 1:5000), eculizumab significantly decreases C5 concentration in the CSF [73]. Moreover, due to a rapid decrease of the complement activity it could be effective and especially attractive during the acute stage of diseases, associated with complement activating IgG1-Abs. Main safety concerns include risk of infectious, particularly with encapsulated bacteria. Eculizumab therapy is associated with a 1000-fold to 2000-fold increased incidence of meningococcal disease, despite tetravalent meningococcal vaccination [74].

Inebilizumab is an afucosylated monoclonal humanized IgG1, targeting CD19 of B-cells. The absence of fucose results in increased antibody dependent cellular cytotoxicity [75]. Potential advantages of anti-CD19 compared to anti-CD20 therapy are the depletion of later stage CD20-negative Abs-producing B-cells, including plasmablasts and some plasma cells. Preclinical data support greater effect of anti-CD19 therapy on B-cell driven autoimmunity [76]. Also, the aforementioned subpopulation of NMOSD-specific plasmablasts express CD19, but not CD20 [42]. The possibility to translate this extended spectrum of targeted B-cells into additional clinical benefits without a higher risk of serious infections must be addressed in further clinical trials. Inebilizumab demonstrated an acceptable safety profile in a phase I study in RRMS and systemic sclerosis and detailed results of the positive phase II/III study in NMOSD are not published yet [77, 78].

## Conclusion

In summary, both localization and function of the target antigen as well as predominant IgG-subtype and/or involvement of T-cell autoimmunity determine therapy responsiveness and outcome in Abs-mediated neurological syndromes. Treatment should be started as early as possible and an increased diagnostic vigilance is required. In refractory cases prompt therapy escalation should be considered. Individual treatment decisions, including immunotherapies established in rheumatologic or oncological diseases, can result in breakthrough improvement. In case of intracellular antigen localization outcome remains poor, therefore new treatment strategies are urgently needed.

## Data Availability

Not applicable.

## Abbreviations

Abs: autoantibodies
AQP4: aquaporin4
CASPR2: contactin associated protein 2
CNS: central nervous systems
CT: computer tomography
DC: dendritic cells
DNER: delta notch-like epidermal growth factor-related receptor
FDG-PET: fluorodeoxyglucose positron emission tomography
GAD: glutamate decarboxylase
IA: immunoadsorption
IL: interleukin
IV: intravenous
IVIG: intravenous immunoglobulin G
LGI1: leucine rich glioma inactivated 1
MOG: myelin oligodendrozyten glycoprotein
MuSK: muscle-specific tyrosine kinase
NF-kB: nuclear factor kB
NF-kB: nuclear factor kB
NMDAR: N-methyl-D-aspartate receptor
NMOSD: neuromyelitis optica spectrum disorder
PE: plasma exchange
PNS: paraneoplastic syndromes
TNF-α: tumor necrosis factor alpha
